# Evaluation of L-carnitine’s protective properties against AlCl_3_-induced brain, liver, and renal toxicity in rats

**DOI:** 10.1371/journal.pone.0317939

**Published:** 2025-01-24

**Authors:** Haifa Ali Alqhtani

**Affiliations:** Department of Biology, College of Science, Princess Nourah Bint Abdulrahman University, Riyadh, Saudi Arabia; Benha University, EGYPT

## Abstract

A common heavy metal in many facets of daily life is aluminum (AlCl_3_), which can be found in food, toothpaste, cosmetics, food additives, and numerous pharmaceutical items. The hippocampus, liver, and kidneys have the highest concentrations of this powerful neurotoxin, which also accumulates over time and contributes to the development of a number of cognitive disorders. Long-term overconsumption of AlCl_3_ results in hepatic and renal toxicity as well as neuronal inflammation. The purpose of the research is to assess the potential protective effects of various L-carnitine dosages as an antioxidant against hebato, renal, and neuronal toxicity in rats caused by aluminum chloride (AlCl_3_) (20 mg/kg, 1/20 LD 50). Six groups (n = 6), consisting of 36 adult albino rats, were randomly assigned. Saline was administered to the control group (GI) by injection. (GII) had given an injection of L-carnitine at a low-dose of 75 mg/kg body weight. An injections of L-carnitine at a high-dose (150 mg/kg) were given to (GIII), and AlCl_3_ (20 mg/kg) was given to (GIV). (GV) administered with L-carnitine (75 mg/kg) and AlCl_3_ (20 mg/kg) by injection. For 60 days, AlCl_3_ (20 mg/kg) and L-carnitine (150 mg/kg) were administered to GVI by injection. Furthermore, the histological structure of the cortex, hippocampus, and hepatic renal tissues appeared to change in response to AlCl_3_. L-carnitine therapy lessened the negative effects of AlCl_3_. The observable improvement in the tissues of the brain, liver, and kidneys further supported this histopathologically. It is possible to draw the conclusion that L-carnitine holds promise as a corrective measure for AlCl_3_, which causes renal toxicity and neural hepatotoxicity in rats. When it comes to adult albino rats, L-carnitine has a negative impact and exhibits ameliorative effects against aluminum chloride.

## Introduction

One of the most prevalent elements in the earth’s crust is aluminium (Al) [1]. It is widely utilised in contemporary daily life and is widely dispersed throughout the environment. The environment and food both contain aluminium that enters the body [2]. Corn, yellow cheese, salts, spices, herbs, tea, and cosmetics like deodorant and antiperspirant formulations are the main foods that contain AlCl_3_ [3]. Furthermore, according to [4–6], it is included in certain medications like antacids, buffered aspirin, anti-diarrheal products, vaccines, and allergen injections. Additionally, it is utilized as a constituent of veterinary medicine, glues, and disinfectants [7]. When drinking water is being purified, a lot of Al sulphate is used as a coagulant agent [8]. Due to aluminum compounds’ detrimental effects on the central nervous system [9], hematology and energy metabolism [10,11], fertility and reproduction [12,13], embryo toxicity, and teratogenicity potency [14], more attention has recently been paid to these compounds. In humans, aluminum can be absorbed and accumulated through food, water, or fruit juices. In healthy individuals, this results in a significant increase in urinary excretion of aluminum as well as gastrointestinal absorption [15].

Methionine and lysine combined to form the amino acid L-carnitine (LC), also known as β-hydroxyl-γ-trimethylamino-butyric acid. According to [16], LC aids in the metabolism of branched-chain amino acids and encourages the oxidation of long-chain fatty acids, which stabilizes cell membranes. Humans obtain it primarily from exogenous sources such as animal diets; secondary sources include methionine and lysine, which are present in smaller concentrations in the liver, kidney, and brain [17]. By making it easier for long-chain fatty acids to enter mitochondria, it makes a substantial contribution to the metabolism of cellular energy [18]. It has demonstrated the ability to scavenge reactive oxygen species (ROS) and function as an antioxidant. Furthermore, it may stabilize damaged cell membranes and prevent mitochondrial oxidative stress caused by mitochondrial damage and apoptosis in various cell types [19,20]. It might be protective against cardio toxicity because of its ant apoptotic and antioxidant qualities [21]. The profile of activities of this molecule indicates that it might be able to lessen the adverse effects of certain pesticides [22]. The endogenous cellular antioxidant defense system inhibits the production of reactive oxygen species (ROS), which can be caused by both exogenous agents and endogenous processes. Oxidative stress results from ROS production exceeding the capacity of the cellular endogenous antioxidant defense system [23]. ROS can harm biochemical macromolecules such as proteins, lipids, and nucleic acids. The purpose of this study was to examine the protective properties of L-carnitine as an antioxidant against AlCl_3_-induced brain, liver, and renal toxicity in albino rats.

## Materials and methods

### Experimental animal

A study was conducted on 36 healthy albino rats weighing 150–155 g. All the animals were given two weeks to acclimatize. The animals were housed in plastic cages with a 12-hour light/dark cycle and a temperature of 25°C. Every day, the health of the animals was examined, and they were provided with conventional food and water without limits. Every attempt has been made to minimize animal suffering and to use as few animals as possible in order to generate trustworthy scientific data. Sigma Chemical Co. (St Louis, Mo., USA) provided the aluminum chloride (AlCl_3_) [24]. We bought L-carnitine from the Arab Company for Pharmaceutical and Medicinal Plants (MEPACO), located in Cairo, Egypt. The 350 mg of L-carnitine that each capsule contained was ground into powder and dissolved in distilled water before being given orally via stomach tube every day at doses of 75 and 150 mg/kg of body weight. Kim et al [25] state that rats were given oral doses of L-carnitine (75 and 150 mg/kg) on a daily basis. This study involving animal participants was approved by the Committee of Health Research Ethics, Deanship of Scientific Research, Qassim University, with ethical committee approval IP (IRP No. 23-41-14).

### Experimental protocol

Six sets of thirty-six rats each were created as follows: Saline was given to Group (GI), the normal control group, 75 mg/kg of L-carnitine (low-dose L-carnitine) was given to Group (GII) of rats, and 150 mg/kg of L-carnitine (high-dose L-carnitine) was given to Group (GIII) of rats. Rats in Groups (GIV) and (GV) received oral injections of AlCl_3_ (20 mg/kg) and L-carnitine 75 mg/kg (low-dose L-carnitine), respectively. Group (GVI): rats were given 150 mg/kg of L-carnitine (high-dose L-carnitine) and 20 mg/kg of AlCl_3_. For two months, AlCl_3_ and L-carnitine were given to each group once daily [26,27].

### Sample collection

Methods of Sacrifice: To obtain blood samples, rats were beheaded 24 hours following their most recent treatment. Following blood collection, the anaesthetized animals were subjected to cervical dislocation as a backup technique to verify death. Following the guidelines set forth by Qassim University Veterinary Hospital, animal disposal was carried out under veterinary supervision. Anaesthesia and/or Analgesia Techniques: To reduce pain and stress during the decapitation procedure, rats were given isoflurane anaesthesia prior to the procedure.

Attempts to Reduce Suffering: Throughout the experiment, the animals received the proper attention, which included enough food, water, and hygienic housing. Prioritizing humane care, the animals were put to sleep under veterinary supervision while adhering to moral guidelines to reduce their suffering. Animal waste and carcasses were disposed of safely and in accordance with established procedures.

Blood was taken from every rat and centrifuged for ten minutes at 3000 rpm after the rats were anesthetized and decapitated. After the liver and kidney were removed and freed from adhering connective tissue, the serum and plasma samples were kept at 0°C until their biochemical analysis the following day. The liver, kidney, and brain were then fixed in 10% formalin for histopathological and immunohistochemical examination of the neural tissue.

According to Zilva [28], the serum concentrations of SGPT and SGOT were obtained kinetically. Calculating the creatinine level in serum involves forming a colorful complex through the reaction of creatinine with picrate in an alkaline medium. Bartels [29] and Fabiny [30] state that interferences are avoided by measuring the complex formation rate quickly. Using reagent kits purchased from BioSystems Chemical Company (Spain). According to the instructions included with the Diamond Diagnostics kits, the blood urea nitrogen (BUN) levels in serum were measured.

### Histological examination

Tissue specimens were fixed in 10% formalin for 24 hours, dehydrated with alcohol, cleared in xylene, and infiltrated with paraffin. They were embedded in paraffin wax and then processed using a rotary microtome to create paraffin sections sectioned into 4–6 μm thick slices. Hematoxylin and eosin staining was applied to visualization, and sections were mounted in DPX for a light microscopic examination [31].

### Statistical analysis

All data from this study were collected and statistically analyzed using the IBM SPSS Statistics software version 27.0 for Windows, USA. The Shapiro-Wilk test was used for the test of data normality. For normally distributed data, the one-way ANOVA test was used to compare more than 2 independent groups, and the Duncan test was used to detect pair-wise comparison. For non-normally distributed data, the paired sample t-test was used to compare 2 paired groups and the Kruskal-Wallis test to compare more than 2 groups. Mann-Whitney test was used to detect pair-wise comparison. The significance of the results was done using a 0.05 level of significance. Results are presented as mean ± SEM or mean rank. Letters from (^a^ to ^f^) meaning the significant letters resulting from the post hoc test (Duncan) in the significant case between treatment means according to ANOVA test or statistical test.

## Results

### Hematological analysis

Compared to the control group, the platelet count in AlCl_3_ showed 483.35 to 351.90 x 10^3^ /cm and the HB concentration ranged from 13.41 to 11.54 g/dl. The HB content, and platelets, respectively, was observed in the other hand-treated groups with L-carnitine (Table 1(. When comparing the control group to the AlCl_3_ group, WBCs increased from 49.55 to 63.50%. But groups receiving treatment for L-carnitine showed a corresponding decrease (Table 1).

**Table 1:** Mean ± SD values of blood of traits as affected by AlCl_3_ individually or co-administered with L-carnitine.

Treatments	HB g/dl	TLC 10^3^ /cm	PLT 10^3^ /cm	Segment %	Staph %	Monocytes %	Lymphocytes %	Eosinophil%
Control	13.41±0.21 ^b^	8.37± 0.035 ^d^	483.35± 3.75 ^b^	43.15±0 .05 ^b^	2.05± 5.20 ^b^	3.30± 0.10 ^a^	49.25±0.05 ^d^	2.25±0.05 ^c^
Low-dose L-carnitine	13.77±0.006 ^a^	8.37± 0.058 ^d^	469.70± 0.30 ^c^	46.70±0.50 ^a^	2.35± 0.05 ^a^	3.70± 0.10 ^a^	45.10±0.30 ^e^	2.15±0.15 ^c^
High-dose L-carnitine	13.99± 0.11 ^a^	8.17± 0.03^e^	440.85± 0.75 ^e^	38.45± 0.05 ^d^	2.15±0.05 ^b^	2.90±0.10 ^b^	42.35±0.05 ^f^	2.45±0.05 ^b^
AlCl_3_ (20 mg/kg)	11.54± 0.34 ^d^	18.26± 0.18 ^a^	351.90± 1.30 ^f^	30.55±0.55 ^f^	1.05± 0.05 ^e^	2.05±0.05 ^c^	63.50±0.50 ^a^	2.85± 0.15 ^a^
AlCl_3_+ low-dose L-carnitine	12.40± 0.10 ^c^	10.85± 0.03 ^c^	457.50±0.50 ^d^	37.15±0.45 ^e^	1.55± 0.05 ^d^	3.70± 0.50 ^a^	55.15± 0.05 ^b^	2.45±0.05 ^b^
AlCl_3_+ high-dose L-carnitine	12.70± 0.06 ^c^	12.21± 0.06 ^b^	500.10± 0.10 ^a^	40.30±0.10 ^c^	1.75±0.05 ^c^	3.55±0.05 ^a^	52.25±0.05 ^c^	2.15±0.05 ^c^
Sig.	0.0001	0.0001	0.0001	0.0001	0.0001	0.0001	0.0001	0.0001

Mean values with small superscripts in the same column were significantly different at *P≤0*.*05*. Small letters from (^a^ to ^f^) meaning the significant letters resulting from the post hoc test (Duncan) in the significant case between treatment means according to ANOVA test or statistical test.

### Liver functions

**Fig 1:**
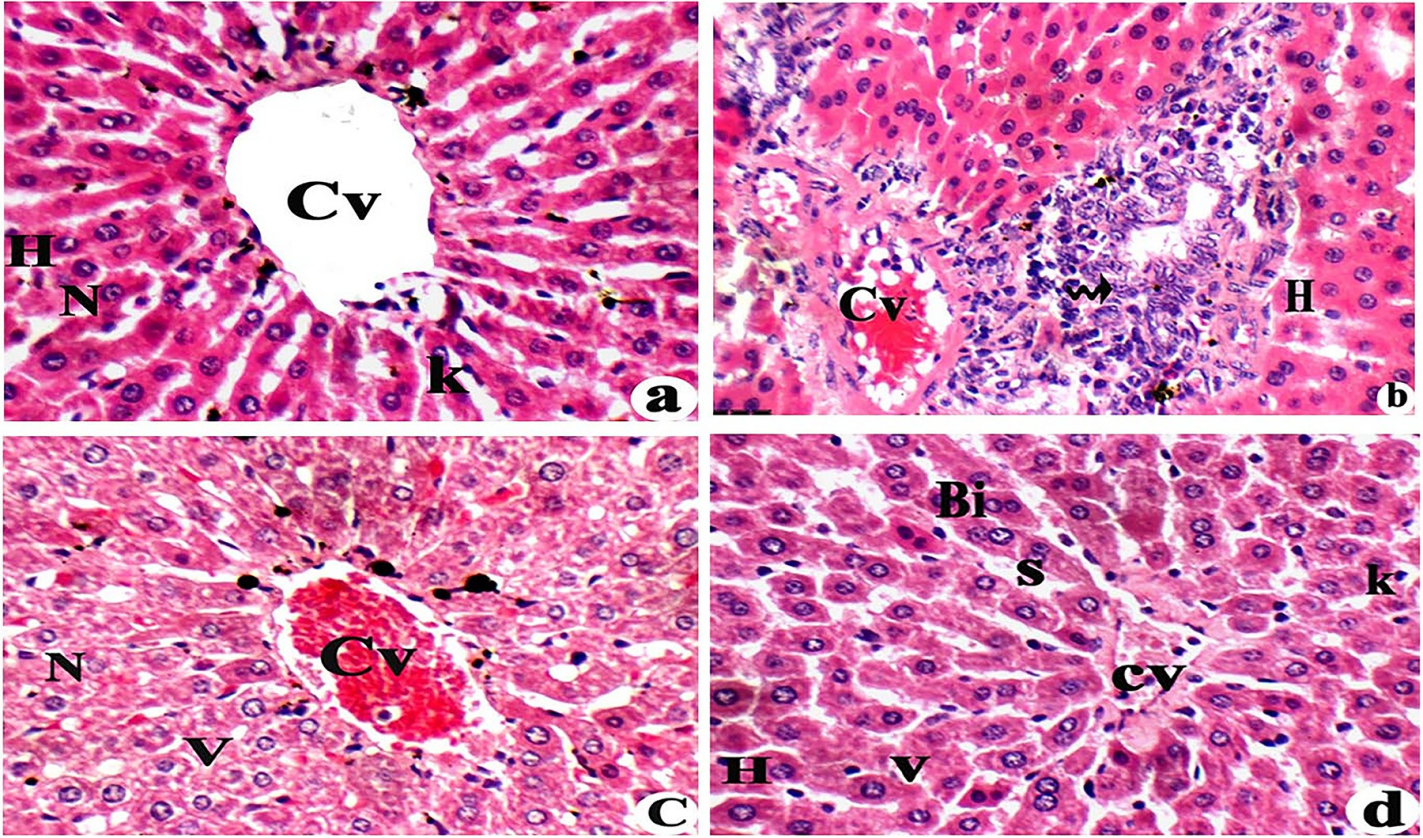
Effects of L-Carnitine on pathological changes in liver tissues of rats exposed to aluminium chloride (AlCl_3_): (a) Experimental controls, (b) AlCl_3_ group, (c) AlCl_3_+ low-dose L-carnitine, and (d) (AlCl_3_+ high-dose L-carnitine). H&E 400 x. Central vein (Cv), hepatocytes (H), nucleus (N), kupffer cells (K), vacuolated cytoplasm (V), massive inflammation (arrow), binucleated hepatocytes (Bi).

When compared to the control group, the ALT and AST concentrations in the AlCl_3_ group were 40.59 to 124.41 U/L and 62.60 to 243.67 U/L increasing, but in the AlCl_3_ treated groups (AlCl_3_+ low-dose L-carnitine), (AlCl_3_+ high-dose L-carnitine) groups 82.97, 66.22 U/L and 207.67, 197.46 U/L, the ALT and AST concentrations remained unchanged as displayed in (Fig 1 and Table 2). The liver functions (ALT and AST) of the various experimental animals are shown in Table 2.

**Table 2:** Mean ± SD values of ALT and AST of traits as affected by AlCl_3_ individually or co-administered with L-carnitine.

Treatments	ALT (U/L)	AST (U/L)
Control	40.59 ± 1.35 ^d^	62.60 ± 4.93 ^c^
Low-dose L-carnitine	40.03 ± 0.62 ^d^	58.03 ± 0.78 ^c^
High-dose L-carnitine	37.68 ± 1.11 ^d^	55.80 ± .40 ^c^
AlCl_3_ (20 mg/kg)	124.41 ± 7.24 ^a^	243.67 ± 10.02 ^a^
AlCl_3_+ low-dose L-carnitine	82.97 ± 5.32 ^b^	207.67 ± 10.79 ^b^
AlCl_3_+ high-dose L-carnitine	66.22 ± 7.75 ^c^	197.46 ± 3.33 ^b^
Sig.	0.0001	0.0001

Mean values with small superscripts in the same column were significantly different at *P≤0*.*05*. Small letters from (^a^ to ^f^) meaning the significant letters resulting from the post hoc test (Duncan) in the significant case between treatment means according to ANOVA test or statistical test.

Effects of L-Carnitine on pathological changes in liver tissues of rats exposed to aluminum chloride (AlCl_3_). Experimental controls showed middle central vein (Cv) with radiating hepatocytes (H) with spherical nucleus (N) and kupffer cells (K) (Fig 1A), AlCl_3_ group showed massive congested, thick central vein (CV) with massive inflammation (arrow) and degenerated hepatocytes (H) (Fig 1B), AlCl_3_+ low-dose L-carnitine showed, some improvements as decreasing central vein congestion (Cv) but hepatocytes with vacuolated cytoplasm (V) (Fig 1C) and (AlCl_3_+ high-dose L-carnitine) showed relative normal hepatocytes (H) structure with increasing binucleated hepatocytes (Bi) and little hepatocytes with vacuolated cytoplasm (V) (Fig 1D).

### Kidney functions

**Fig 2:**
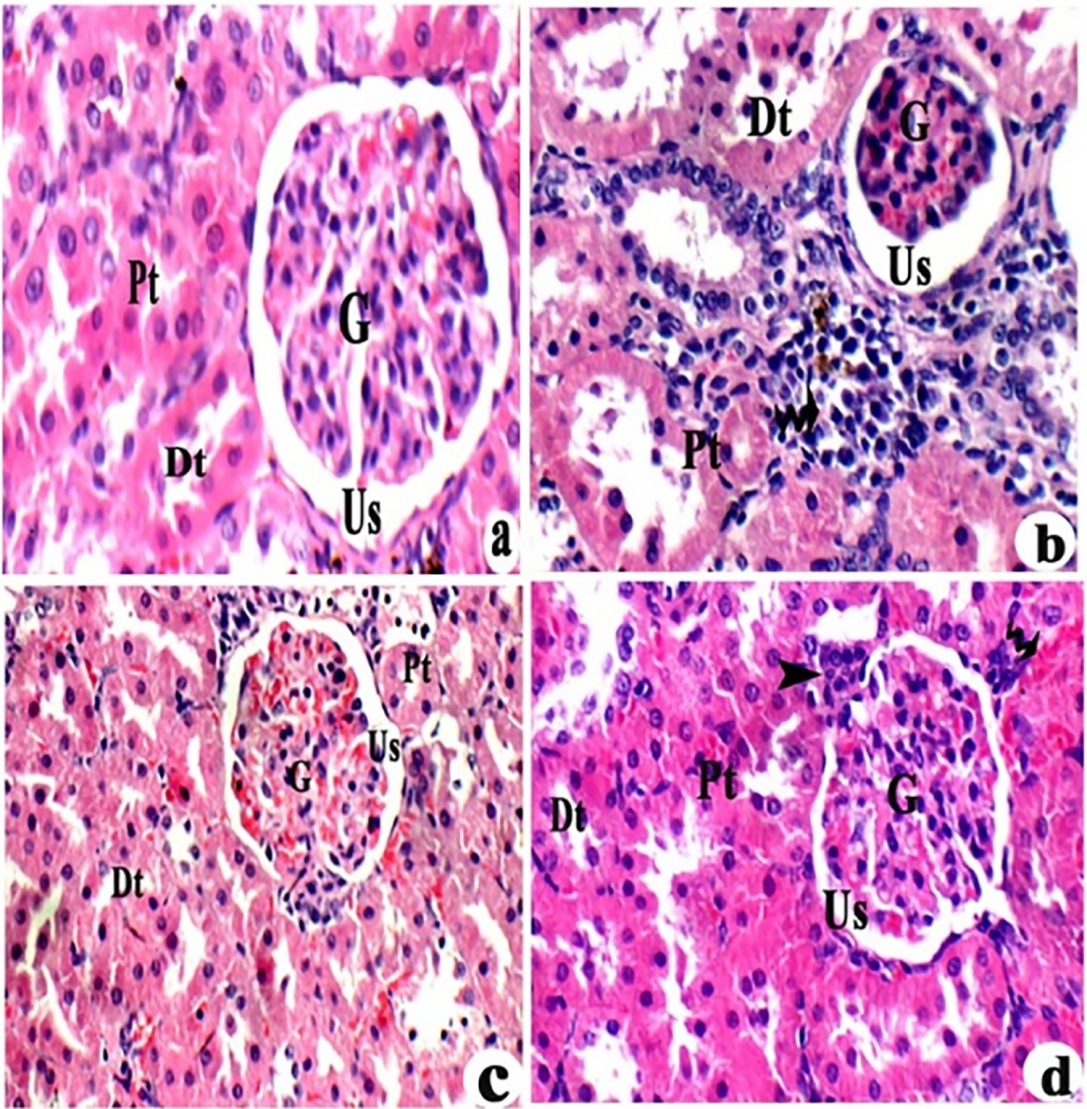
Effects of L-Carnitine on pathological changes in kidney of rats exposed to aluminium chloride (AlCl_3_): (a) Experimental controls, (b) AlCl_3_ group, (c) AlCl_3_+ low-dose L-carnitine, and (d) (AlCl_3_+ high-dose L-carnitine). H&E 400 x. Glomerulus (G), Urinary space (Us),), massive inflammation (arrow), proximal (Pt), distilled tuples (Dt), little congestion (bent arrow) & inflammation (head arrow).

In the AlCl_3_ group, the creatinine and blood urea concentrations increased from 0.75 to 0.96 mg/dL and from 30.39 to 57.90 mg/dL compared to the control group. However, in the AlCl3 treated groups (AlCl_3_+ low-dose L-carnitine), (AlCl_3_+ high-dose L-carnitine) groups, 0.85, 0.82 mg/dL and 39.68, 39.80 mg/dL, the creatinine and blood urea concentrations change as shown in (Table 3 and Fig 2). Table 3 and Fig 2 display the different experimental animals’ kidney functions (creatinine and blood urea).

**Table 3:** Mean ± SD values of creatinine and blood urea of traits as affected by AlCl_3_ individually or co-administered with L-carnitine.

Treatments	Creatinine (mg/dL)	Blood Urea (mg/dL)
Control	0.75 ± 0.02 ^d^	30.39 ± 0.59 ^c^
Low-dose L-carnitine	0.73 ± 0.01 ^d,e^	29.63 ± 1.03 ^c,d^
High-dose L-carnitine	0.72 ± 0.01 ^e^	28.29 ± 0.63 ^d^
AlCl_3_ (20 mg/kg)	0.96 ± 0.02 ^a^	57.90 ± 0.10 ^a^
AlCl3+ low-dose L-carnitine	0.85 ± 0.02 ^b^	39.68 ± 0.42 ^b^
AlCl3+ high-dose L-carnitine	0.82 ± 0.02 ^c^	39.80 ± 1.40 ^b^
Sig.	0.0001	0.0001

Mean values with small superscripts in the same column were significantly different at *P≤0*.*05*. Small letters from (^a^ to ^f^) meaning the significant letters resulting from the post hoc test (Duncan) in the significant case between treatment means according to ANOVA test or statistical test.

Effects of L-Carnitine on pathological changes in kidney of rats exposed to aluminum chloride (AlCl_3_). Experimental controls showed normal glomerulus (G) with regular urinary space (Us),normal proximal (Pt) and distilled tuples (Dt) (Fig 2A). AlCl_3_ group showed atrophied and congested glomeruli (G) with irregular urinary space(Us), massive inflammation (arrow) and degenerated tuples (Fig 2B). AlCl_3_+ low-dose L-carnitine showed, some improvements as decreasing congested glomeruli (G) (Fig 2C). AlCl_3_+ high-dose L-carnitine showed relative normal glomeruli (G) and urinary space (Us) with little congestion (bent arrow) & inflammation (head arrow) in parenchymal tissue (Fig 2D).

### The brain histopathological

**Fig 3:**
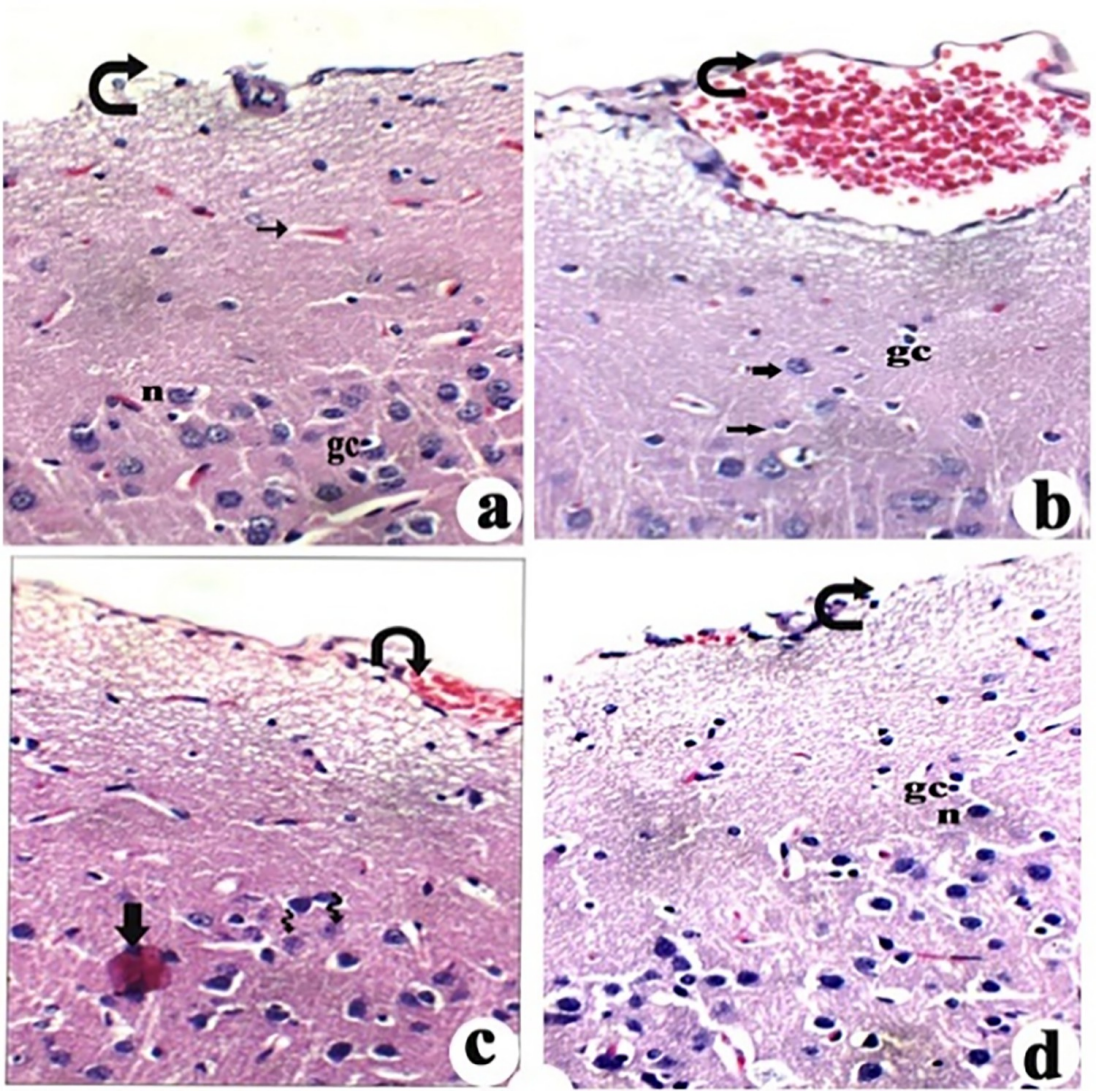
Effects of L-Carnitine on pathological changes in cortex of the brain of rats exposed to aluminium chloride (AlCl_3_): (a) Control brain, (b) AlCl_3_ group, (C) AlCl_3_ treated with low-dose L-carnitine, and (d) AlCl_3_ treated with high-dose L-carnitine. (H&E X 400). Neurons (n), glial cells (gc), sub-meningeal edema (bent arrow), degenerated neurons (thick arrow), picnotic neurons (thin arrow), plaque-like area (thick arrow), little congestion (bent arrow).

Effects of L-Carnitine on pathological changes in cortex of the brain of rats exposed to aluminum chloride (AlCl_3_). Control brain showed average meninges (bent arrow) with average sub-meningeal blood vessels (arrow), cerebral cortex with average neurons (n), average glial cells (gc) (Fig 3A). AlCl_3_ group showed meninges with mild sub-meningeal edema (bent arrow), markedly scattered degenerated neurons (thick arrow) and picnotic neurons (thin arrow) and glial cell (gc) (Fig 3B). AlCl_3_ treated with low-dose L-carnitine showed cerebral cortex with average meninges with mildly congested (bent arrow), cerebral cortex with average degenerated neurons (thin arrow), average glial cells and average intra-cerebral blood vessels, eosinophilic plaque-like area (thick arrow) (Fig 3C). AlCl_3_ treated with high-dose L-carnitine showed cerebral cortex with average meninges with little congestion (bent arrow), cerebral cortex with average neurons (n), average glial cells (gc) (Fig 3D).

**Fig 4:**
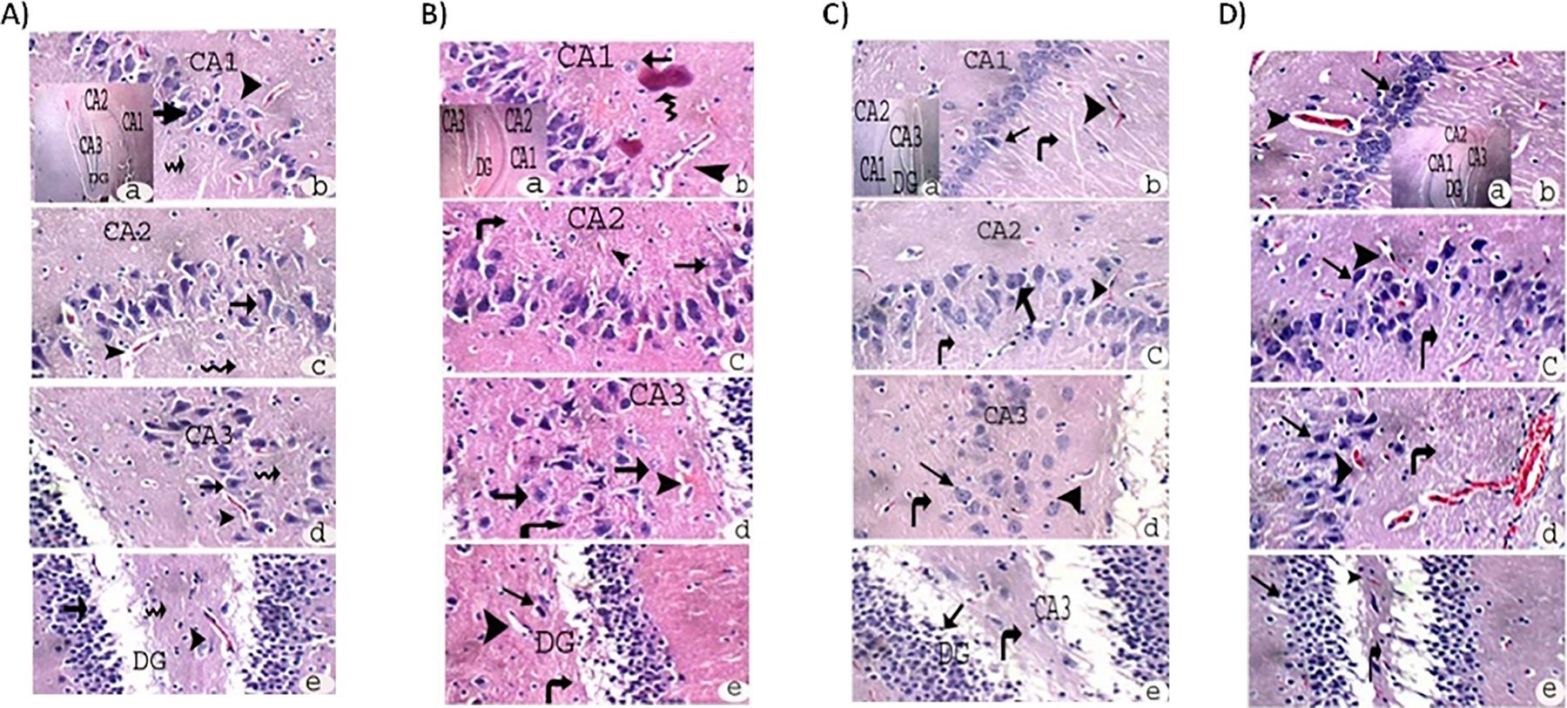
Complete hippocampus of experimental A); Control, B); AlCl_3_ group, C); AlCl_3_+ low-dose L-carnitine, and D); AlCl_3_+ high-dose L-carnitine group showed average blood vessels (head arrow) in Cornu Amonis (CA1), (CA2), (CA3), and in (DG). H&E X (a) = 100 and (b, c, d, e = 400). Pyramidal neurons (arrow), interneuron area (zigzag arrow), average blood vessels (head arrow), cornu Ammonis (CA1, CA2, CA3) and dentate gyrus (DG).

The hippocampus of the experimental control group (Fig 4A), AlCl₃ group (Fig 4B), AlCl₃+ low-dose L-carnitine group (Fig 4C), and AlCl₃+ high-dose L-carnitine group (Fig 4D) showed average pyramidal neurons (arrow), average interneuron area (zigzag arrow), and average blood vessels (head arrow) in the Cornu Ammonis (CA1, CA2, CA3) and dentate gyrus (DG).

### The brain immunohistochemical

**Fig 5:**
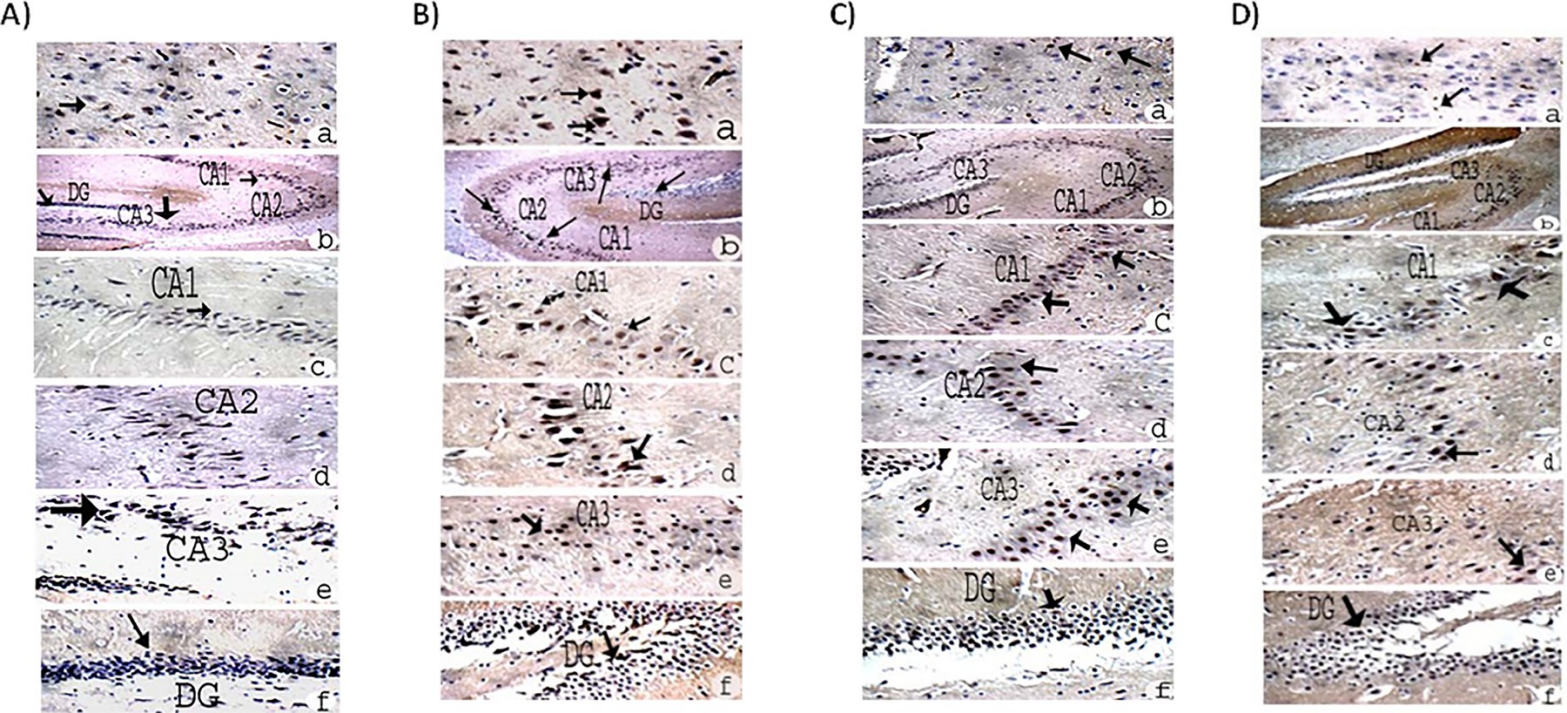
Experimental A); Control, B); AlCl_3_ group, C); AlCl_3_+ low-dose L-carnitine group, and D); AlCl_3_+ high-dose L-carnitine group showed: **Cortex** and complete hippocampus of caspase 3: a): Cortex showed negative cytoplasmic reactivity (arrow) for Caspase-3 in cerebral cortex, (b, c, d, e, f): Showed Hippocampus with negative cytoplasmic reactivity (arrow) for Caspase-3 in CA1, CA2, CA3, and in DG H&E. a, c, d, e. f. = 400x and a = 200x.

Caspes expression was considered as cytoplasmic staining. The expresstion as classified as negative (o), weak(+), moderate (++) & marked (+++) as showed in (Fig 5 and Tables 4 and 5). Experimental Control (Fig 5A), AlCl_3_ group (Fig 5B), AlCl_3_ with low-dose L-carnitine group (Fig 5C), AlCl_3_ with high-dose L-carnitine group (Fig 5D) showed cortex and complete hippocampus of caspase 3 a): cortex showed negative cytoplasmic reactivity (arrow) for Caspase-3 in cerebral cortex, (b,c, d, e, f): showed Hippocampus with negative cytoplasmic reactivity (arrow) for Caspase-3 in CA1, CA2, CA3, and in DG.

**Table 4:** Mean values of percentage of reactive protein according to different treatments in various locations in the brain and hippocampus sites.

Treatments	Brain	Hippocampus			
	Cortex	CA1	CA2	CA3	DG
**Control**	100% ^c^ (0)	100% ^d^ (0)	100% ^c^ (0)	100% ^c^ (0)	100% ^c^ (0)
**Low-dose L-carnitine**	100% a (+++)	100% ^a^ (+++)	100% ^a^ (+++)	100% ^a^ (+++)	100% ^a^ (+++)
**High-dose L-carnitine**	100% ^b^ (+)	100% ^b^ (++)	100% ^a^ (+++)	100% ^a^ (+++)	100% ^b^ (+)
**AlCl**_**3**_	100% ^b^ (+)	100% ^c^ (+)	100% ^b^ (++)	100% ^b^ (++)	100% ^b^ (+)
**AlCl**_**3**_ **+ low-dose L-carnitine**	100% ^c^ (0)	100% ^d^ (0)	100% ^c^ (0)	100% ^c^ (0)	100% ^c^ (0)
**AlCl**_**3**_ **+ high-dose L-carnitine**	100% ^c^ (0)	100% ^d^ (0)	100% ^c^ (0)	100% ^c^ (0)	100% ^c^ (0)
**Sig.**	0.004				

Percentages with small letters (a, b, c, d) in the same column are significantly different at P≤0.05

(0) = No reaction, (+) = Weak reaction, (++) = Moderate reaction, (+++) = Marked reaction, AlCl3 = Aluminum Chloride

**Table 5:** Mean values of percentage of reactive protein in the different hippocampus locations in each treatment separately.

Treatments	Hippocampus				Sig.
	CA1	CA2	CA3	DG	
**Control**	100% ^a^ (0)	100% ^a^ (0)	100% ^a^ (0)	100% ^a^ (0)	0.999
**Low-dose L-carnitine**	100% ^a^ (+++)	100% ^a^ (+++)	100% ^a^ (+++)	100% ^a^ (+++)	0.999
**High-dose L-carnitine**	100% ^b^ (++)	100% ^a^ (+++)	100% ^a^ (+++)	100% ^c^ (+)	0.012
**AlCl**_**3**_	100% ^b^ (+)	100% ^a^ (++)	100% ^a^ (++)	100% ^b^ (+)	0.012
**AlCl**_**3**_ **+ low-dose L-carnitine**	100% ^a^ (0)	100% ^a^ (0)	100% ^a^ (0)	100% ^a^ (0)	0.999
**AlCl3+ high-dose L-carnitine**	100% ^a^ (0)	100% ^a^ (0)	100% ^a^ (0)	100% ^a^ (0)	0.999

Percentages with small letters (a, b, c) in the same row are significantly different at P≤0.05

(0) = No reaction, (+) = Weak reaction, (++) = Moderate reaction, (+++) = Marked reaction, AlCl3 = Aluminum Chloride

## Discussion

Numerous organ systems may be harmed when toxic metals are exposed to human populations. Aluminum (AlCl_3_) is a frequently studied toxic metal that has been linked to numerous illnesses [32]. AlCl_3_, the third most common element, makes up about 8% of all the minerals in the crust of the Earth [33]. Dietary aluminum studies carried out in numerous countries have estimated the typical adult intake to be between 3 and 12 mg/day [34]. According to Nehru et al [35], AlCl_3_ is a neurotoxin that is frequently studied and affects the nervous system, including different parts of the brain. According to some specialists, AlCl_3_ penetrates the blood-brain barrier and contributes to the development of neurofibrillary tangles that resemble Alzheimer’s disease [36].

Most, if not all, mammalian tissues, including the brain, naturally contain the compound L-carnitine [37]. Even though the kidney, liver, and brain can synthesize carnitine and obtain it through diet, humans are thought to require this nutrient "conditionally" when intracellular levels are low (e.g., premature infants, elderly patients, diabetes, and genetic conditions resulting in primary or secondary carnitine deficiency) [38]. L-carnitine is widely found in nature, and people are becoming more aware of its possible health advantages. It has been discovered that only L-carnitine is bioactive, speeds up lipid metabolism, and transports activated long-chain fatty acids into the mitochondria. A small, water-soluble molecule crucial for the metabolism of fat in mammals is called L-carnitine [39]. L-carnitine and its derivatives have been shown to exhibit a wide range of biological activities, including an antiperoxidative effect on several tissues and antioxidant and anti-inflammatory effects on various pathophysiological conditions [40,41].

By reducing caspase activity, reducing oxidative stress, and strengthening antioxidant defense systems, L-carnitine can reduce cell apoptosis [42,43]. L-carnitine has been shown to effectively inhibit the cleavage and activation of downstream caspases, thereby suppressing the activity of caspase 8, an initiator caspase, and the processing of caspase 9 [44]. Compared to synthetic inhibitors, which inevitably cause side effects, the use of a straightforward, non-toxic metabolite such as carnitine as an anti-apoptotic agent is highly appealing [44]. L-carnitine has been shown to be able to prevent H_2_O_2_-induced cell death in human proximal tubule epithelial cells (HK-2), reported by Modanloo et al [45]. The findings by Koohpeyma et al [46] demonstrated that the presence of L-carnitine abolished the effects of mitochondrial dysfunction linked to cell apoptosis, including membrane potential loss, up- and down-regulation of Bax and Bcl-2, activation of caspase-3, and release of cytochrome c. Furthermore, in the serum-deprived MC3T3-E1 cells, Xie et al. [47] demonstrated that L-Ca reduced cytochrome c release and caspase-3 and caspase-9 activation.

According to the current study, administering AlCl_3_ causes harm to the kidney, liver, hippocampal, and cortex of the brain. This manifested as shrinking cell bodies with pericellular haloes and damage to neural cells. Additionally, certain pyramidal cells displayed vacuolated neuropil and pyknotic nuclei. This was consistent with earlier research showing a connection between the use of AlCl_3_ and the generation of reactive oxygen species (ROS) and oxidative stress. They continued by saying that because brain tissues use a lot of oxygen, have a lot of polyunsaturated fatty acids in their membranes, and have low levels of antioxidant enzymes, they are particularly vulnerable to the harmful effects of reactive oxygen species (ROS) [48]. Furthermore, AlCl_3_ can interact with the lipids in the plasma membrane to cause lipid peroxidation. According to earlier studies, peroxidation of membrane lipids can lead to an increase in membrane leakage, mitochondrial dysfunction, and damage to DNA, lipids, and proteins, which can ultimately cause cellular degeneration and death [49]. It has been demonstrated that aluminum chloride (AlCl_3_) produces reactive oxygen species (ROS) and inhibits antioxidant enzymes in the brain, testis, liver, kidneys, lungs, and seminal plasma [50,51]. Moreover, ROS participate in lipid peroxidation (LPO), which modifies the function of mitochondria and increases their permeability [52]. AlCl_3_ consumption causes oxidative damage, cell death, and toxicity by upsetting the oxidative/antioxidative equilibrium, causing LPO, and reducing the activities of antioxidant enzymes [53,54].

Free radical production is a component of AlCl_3_-induced oxidative stress [34,55,56]. According to Abdel Moneim [33], the pathophysiology of Parkinson’s disease and Alzheimer’s disease is closely linked to the damage that ROS overproduction causes to neurons. Prior research on AlCl_3_ exposure revealed elevated reactive oxygen species (ROS), which were linked to increased electron chain activity, enhanced mitochondrial activity, and electron leakage. Thus, it is possible to speculate that oxidative stress plays a role in the toxicity caused by AlCl_3_. One of the primary signs of oxidative damage is LPO, which has been linked to the toxicity of numerous toxic metals.

Numerous studies have shown that Al’s in vivo and in vitro toxicities adversely impact macromolecules and cellular structure, resulting in cytotoxicity, the production of reactive oxygen species (ROS), mitochondrial dysfunction, inflammation, cell death, genetic damage, and carcinogenicity [53,54,57]. According to Sun et al [58], in addition to the liver and bone, excessive exposure to Al has harmful effects on the immune, respiratory, neurological, and reproductive systems.

According to Lentini et al [59], only a tiny portion of the aluminum that builds up in the human body through the skin comes from tainted food and water. Nephrotoxicity and renal impairment result from the kidneys quickly eliminating the majority of this Al. Furthermore, Al-Kahtani et al [60]recently discovered that AlCl_3_-induced hepatotoxicity causes oxidative stress and apoptosis in rats. Wang et al [61] also came to the conclusion that AlCl_3_ accumulation in the hepatic tissue causes hepatotoxicity.

Bioactive L-carnitine has been shown to expedite lipid metabolism and function as a shuttle for activated long-chain fatty acids into the mitochondria. An essential component of the metabolism of fat in mammals, L-carnitine is a tiny, water-soluble molecule [39]. The biological activities of L-carnitine and its derivatives are diverse, exhibiting anti-peroxidative effects on multiple tissues [40] as well as antioxidant and anti-inflammatory effects on a variety of pathophysiological conditions [41]. Varcocele is a major cause of male infertility; however, L-carnitine has been shown to improve sperm quality by preventing oxidative damage [62]. Additionally, studies have shown that L-carnitine is beneficial in treating varicocele.

The production of reactive oxygen species (ROS) and the body’s antioxidant defenses are out of balance when oxidative stress occurs, which is a major factor in tissue damage. When too many ROS are produced, the body cannot counteract them, which causes lipid peroxidation, protein oxidation, and DNA fragmentation, which damages cells. This imbalance is a major mechanism behind tissue damage in the brain, kidneys, liver, and other organs. Specifically, exposure to environmental toxicants such as aluminum chloride (AlCl_3_) has been demonstrated to cause excessive production of reactive oxygen species (ROS), which exacerbates oxidative stress and contributes to inflammation and additional tissue degeneration. L-carnitine helps to mitigate this damage by scavenging ROS, restoring cellular redox balance, and preventing further injury. According to recent research, antioxidants protect against oxidative damage and maintain tissue function in toxic environments [63–65].

## Conclusion

This study investigated how adult male albino rats exposed to aluminum chloride (AlCl_3_) responded to L-carnitine. L-carnitine effectively reduced oxidative stress and cellular damage, thereby mitigating the harmful effects of AlCl_3_ on the liver, kidneys, and brain of the rats. The neurotoxicity linked to AlCl_3_ exposure was lessened, and the function of these essential organs was preserved thanks to this protective action. The findings highlight the twofold character of L-carnitine, demonstrating both its protective advantages against AlCl_3_ toxicity. When it comes to adult albino rats, L-carnitine has a negative impact and exhibits ameliorative effects against aluminum chloride.
